# New reports of pathogen spectrum associated with bulb rot and their interactions during the development of rot in tulip

**DOI:** 10.1186/s12863-024-01218-w

**Published:** 2024-05-09

**Authors:** Qadrul Nisa, Gazala Gulzar, Mohammad Saleem Dar, Efath Shahnaz, Saba Banday, Zahoor A. Bhat, Mohamed A. El-Sheikh, Sajad Un Nabi, Vivak M. Arya, Ali Anwar, Sheikh Mansoor

**Affiliations:** 1 Division of Plant Pathology, Sher-e-Kashmir University of Agricultural Sciences & Technology of Kashmir, 190025 Shalimar, Jammu & Kashmir, India; 2 Dryland Agricultural Research Station, 190007 Rangreth, Jammu & Kashmir, India; 3 Botany and Microbiology Department, College of Science, King Saud University, Riyadh-11451, Saudi Arabia; 4 Plant Pathology, Central Institute of Temperate Horticulture, 190007 Jammu & Kashmir, India; 5 Division of Soil Science and Agriculture Chemistry, Sher e Kashmir University of Agricultural Sciences and Technology, Jammu, India; 6 Department of Plant Resources and Environment, Jeju National University, 63243 Jeju, South Korea

**Keywords:** Bulb rot, New report, Pathogen-pathogen interactions, Tulip, Virulence

## Abstract

Bulb rot, a highly damaging disease of tulip plants, has hindered their profitable cultivation worldwide. This rot occurs in both field and storage conditions posing significant challenges. While this disease has been attributed to a range of pathogens, previous investigations have solely examined it within the framework of a single-pathogen disease model. Our study took a different approach and identified four pathogens associated with the disease: *Fusarium solani, Penicillium chrysogenum, Botrytis tulipae, and Aspergillus niger*. The primary objective of our research was to examine the impact of co-infections on the overall virulence dynamics of these pathogens. Through co-inoculation experiments on potato dextrose agar, we delineated three primary interaction patterns: antibiosis, deadlock, and merging. In vitro trials involving individual pathogen inoculations on tulip bulbs revealed that *B. tulipae*,was the most virulent and induced complete bulb decay. Nonetheless, when these pathogens were simultaneously introduced in various combinations, outcomes ranged from partial bulb decay to elongated rotting periods. This indicated a notable degree of antagonistic behaviour among the pathogens. While synergistic interactions were evident in a few combinations, antagonism overwhelmingly prevailed. The complex interplay of these pathogens during co-infection led to a noticeable change in the overall severity of the disease. This underscores the significance of pathogen-pathogen interactions in the realm of plant pathology, opening new insights for understanding and managing tulip bulb rot.

## Introduction

Tulip (*Tulipa* spp.) the premier ornamental flowering bulb, belongs to the Genus *Tulipa*, family Liliaceae. It is the first ranking bulbous ornamental plant in the world and has gained popularity due to its beauty and economic value [1]. It is consistently ranked behind rose, chrysanthemum and carnation in the world cut flower trade but is ahead of gladiolus in Netherlands. The tulip, which is native to Central Asia was likely to be first cultivated in Persia in the 12th century [2, 3]. After that they were finally introduced to Netherlands in 1571 via Turkey [4]. Tulips grow successfully in hilly states of India like Jammu and Kashmir and Himachal Pradesh, but unsatisfactorily on the plains. In Himachal Pradesh, premium cut flowers are grown in glass greenhouses, while field planting of bulbs is more cost-effective. Kashmir valley with its ideal climatic conditions and soil fertility has a tremendous potential for tulip production, the evidence of which is the establishment of Indra Gandhi Memorial Tulip Garden in Srinagar which is the largest tulip gardens in Asia, extending on 40 ha of land [1]. Opening of tulip garden in Kashmir has preponed tourist arrival by over a month thus boosting states economy directly and indirectly. Moreover, a perfect tulip bloom every spring has become a point of prestige for Kashmir valley.

Tulip is affected by fungal, bacterial and viral diseases [5]. Among fungal diseases it is affected by bulb rot (*Fusarium* spp., *?talisize Pythium* spp.), crown rot (*Corticium rolfsii*), Pythium root rot, soft rot (*Pythium ultimum* Trow.), etc. [6–9] but bulb rot is one of the most destructive diseases and has alarmed the tulip growers throughout the world [10]. The disease both in storage and field accounts for 20–30% loss of bulbs in tulip garden every year. To compensate for the losses in tulip bulb production, the government annually acquires 4 million to 5 million bulbs from the Netherlands. A series of pathogens have been reported from time to time to be associated with the bulb rot of tulip. Cury first reported the disease in 1931 from Netherlands and identified it as *Fusarium* bulb rot [11]. Lorbeer in 2004 reported *Botrytis* spp. as the causal agent of many diseases like *Botrytis* leaf blight and *Botrytis* neck rot in many bulbous crops like onion, tulip and gladiolus [12]. Many species of *Penicillium* were reported by Overy in 2005 among which *Penicillium hirsutum*, *P. radicicola*, *P. tulipae* and *P. venetum* were predominant bulb rot pathogens of *Tulipa gesneriana* [13]. Also, *Pectobacterium caratovorum* was the first report from Turkey as a bacterial pathogen seen to cause soft rot of tulip bulbs [14]. Similarly isolation trials carried out in Egypt yielded a large number of pathogens viz. *Fusarium oxysporum*, *Fusarium moniliforme*, *Fusarium solani*, *Rhizoctonia tuliparum*, *Aspergillus* spp., *Rhizopus arrizhus* and *Pythium* spp. as the causal pathogens associated with the bulb rot of Tulip and other ornamental crops [15]. In India, the preliminary work on bulb rot was reported from Himachal Pradesh followed by Jammu and Kashmir which identified the causal organism as *Fusarium oxysporum* Schlecht f.sp. *tulipae* Apt. Recently, we reported *F. solani* as one of the causal organisms to be associated with the bulb rot of tulip [1]. In the present manuscript we report *Penicillium chrysogenum, Botrytis tulipae* (Lib.) Comb and *Aspergillus niger* Gams as the causal organisms to be associated with the disease. Many scientists studying tulip bulb rot have mainly focused on individual host-pathogen relationships, overlooking the complexities that arise from multiple species or genotypes [16]. Pathogen-pathogen interaction, is a relatively new but increasingly important field for comprehending and managing microbial diseases [17]. While tulip bulb rot can be caused by various pathogens, there’s a lack of research on how these pathogens interact with each other. To address this gap, our current research aims to isolate and identify the primary fungal pathogens responsible for tulip bulb rot and investigate the nature of interactions between them.

## Materials and methods

The present study was conducted in the Division of Plant Pathology and Division of Biotechnology, Faculty of Horticulture, Sher-e-Kashmir University of Agricultural Sciences & Technology of Kashmir, Shalimar. The details of the materials used and methodology adopted during different experiments to achieve the objectives of the study are described as under.

### Sample collection

**Fig. 1:**
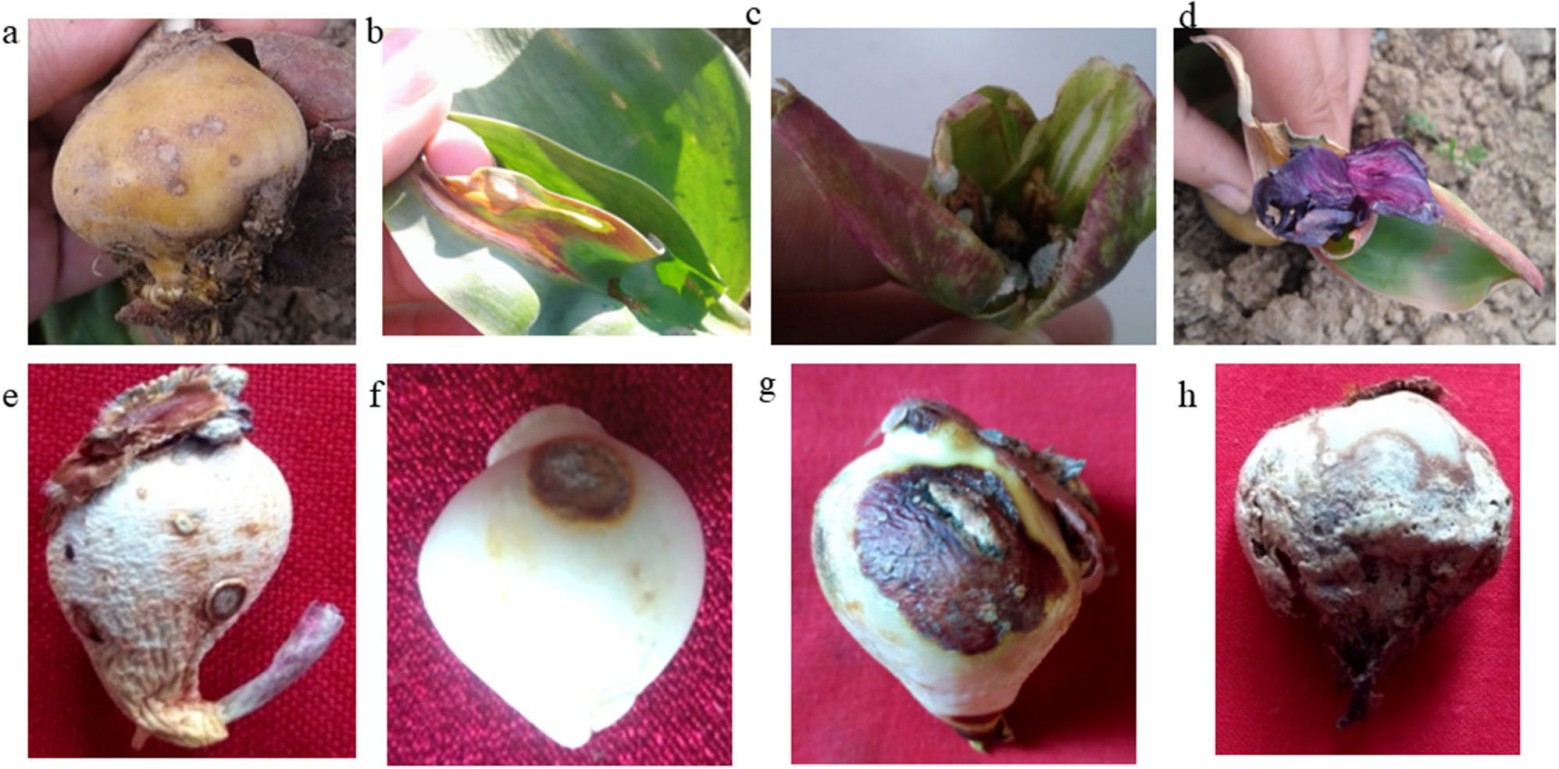
Symptoms associated with bulb rot of Tulip in field conditions and under storage. (**a**) Yellow flecks on outer surface of tulip bulb, (**b**) Characteristic purple coloration on foliage, (**c**) Characteristic purplish coloration on flower bud, (**d**) Deformed or blasted flower, (**e**) lesions on outer surface under storage, (**f**) Sunken yellow spot with initial rot under storage, (**g**) Coalescing of spots and gradual rotting of tulip bulb under storage, (**h**) Final tulip bulb rot and decay under storage

The sample collection was done in 2019 and 2020 during the growth period in the fields as well as under storage conditions. The experimental trial was laid down for the variety Orange Emperor in the field of Plant Pathology and was regularly monitored for the symptoms of bulb rot (Fig. 1). Sample collection was done at three stages of crop, during the growth period in the field (March-April), at the time of harvesting (June) as well as during storage (July-October). 30 samples were taken during growth stage of the plant from the field and 30 during harvesting. After harvesting, the bulbs were kept in mesh bags under storage. Later on 50 samples were also taken from bulbs under storage (20–22 °C with relative humidity of 65%). which showed the symptoms of bulb rot. Healthy bulbs were also procured for pathogenicity and interactional studies.

### Isolation and purification of fungi associated with the bulb rot of tulip

The isolation of the causal agent was done by tissue bit transfer method [18]. Bulbs showing typical symptoms of bulb rot were taken, and diseased portions were cut into small bits (2 mm) with a sharp sterilized blade in such a way that each diseased bit contained a portion of healthy tissue along with it. These bits were subjected to surface sterilization with 0.1 per cent mercuric chloride for 30 s followed by three rinses in sterilized distilled water to remove the traces of mercuric chloride. These bits were then blotter dried and transferred aseptically to potato dextrose agar (PDA) ^o^C medium in sterilized Petri plates and incubated at 25 ± 2oC to maintain its growth for further studies. The plates were observed regularly and mycelial growth on the bits was sub-cultured as soon as the growth appeared. Purification of the isolated fungi was done using single spore isolation method [19]. For this purpose, a spore suspension was evenly spread on the surface of 2 per cent agar medium in Petri plates. The germinating spores along with agar disc were lifted with the help of a sterilized needle, transferred to PDA plates under aseptic conditions and incubated at 25 ± 2^o^C to maintain its growth. The pure culture so obtained was maintained for further study through regular sub-culturing.

### Frequency of fungi associated with the bulb rot of tulip

Isolation frequency of fungi associated with the tulip bulb was calculated as per formula given by Aneja, 2007 [20]:

$$Percent{\text{ }}occurrence = \frac{{No.{\text{ }}of{\text{ }}colonies{\text{ }}of{\text{ }}individual{\text{ }}fungi}}{{Total{\text{ }}no.{\text{ }}of{\text{ }}colonies{\text{ }}of{\text{ }}all{\text{ }}fungi}} \times 100$$

### Pathogenicity tests

The pathogenicity test was conducted under laboratory conditions following pinprick method [1]. Healthy tulip bulbs of susceptible variety Orange Emperor were collected and their outer tunics were removed to check the lesion free bulbs. Then the bulbs were surface sterilized by giving a 3 min dip in 0.1 per cent aqueous mercuric chloride followed by five washings in sterile distilled water. Nine bulbs were taken for each pathogen. Three bulbs were injured with a sterilized teasing needle, three were left uninjured and the remaining three were kept as control. Spore suspension was prepared by suspending freshly growing culture of the respective test pathogens in sterile distilled water, adjusted to a density of 1 × 10^6^ conidia /ml. Both injured and uninjured bulbs were given a 15 min dip in the spore suspension and were placed in moist chamber. The bulbs kept for control were dipped in sterile water. These moist chambers were then incubated at 25 ± 2 ^o^C and regularly observed for appearance of symptoms. After recording the typical symptoms of each test pathogen on the respective bulbs, the pathogens were re-isolated and compared with original culture to satisfy Koch’s postulates.

### Morpho-cultural and molecular characterization

Cultural and morphological characters of the causal pathogens, were studied in the Division of Plant Pathology for their identification. Morpho-cultural characters exhibited by mono-conidial cultures were recorded for shape, colour, size and septation of colonies, mycelium, conidiophores, conidia and sclerotia, wherever formed.

### Molecular characterization

DNA extraction was carried out using 400 µl Sodium dodecyl sulphate (SDS) extraction buffer [21]. Polymerase chain reaction (PCR) amplification for Internal Transcribed Spacer 1 and Internal Transcribed Spacer 4 (ITS1-ITS4) region of all the pathogens and *β-tubulin* gene wherever necessary was carried out in a reaction volume of 25 µl in 0.2 ml PCR microfuge tubes [22, 23]. The amplification reaction was carried out in thermocycler (Applied biosystems, model Veriti) under the following conditions 94ºC for 4 min, 40 cycles of 94ºC for 30s, 72ºC for 1 min and 72ºC for 8 min. The amplified products were electrophoresed on 1 per cent agarose gel in 1x TAE buffer run at 100 V for 45 min and compared with DNA ladder (IKB and 100 bp) as standard. 30 µl amplified PCR product of each respective fungus were put in 1.5 ml microfuge tubes along with 50 µl primer in separate microfuge tubes having a concentration of 10mmol/µl and were out sourced for sequencing to Biokart lab, Bangalore. The sequenced data was analysed using bioinformatics tool NCBI BLAST for comparative sequence analysis between query sequences and data base sequences. The sequences unique to the sample were marked. Sequence similarity with already existing sequence in the GenBank was done using “BLAST N similarity search”. The phylogenetic analysis was performed using the neighbour joining method in Mega-X V-10.0 software.

### In vitro interaction of pathogens associated with the bulb rot of tulip on potato dextrose agar medium

In order to study the interactional behaviour of bulb rotting pathogens, we first inoculated them in different combinations on PDA. The pathogens *Fusarium solani, Penicillium chrysogenum, Botrytis tulipae* and *Aspergillus niger* were grouped in total of ten different combinations in petri plates containing potato dextrose agar. The Petri plates were incubated at 25 ± 2^o^C for 15 days. The single culture of each test pathogen was served as check. Three replications were kept for each combination set. Observations for radial growth inhibition of a particular pathogen as a result of interactions of other pathogen(s) were calculated by using the formula as under [24]:

$$Percent{\text{ }}inhibition = \frac{{colony{\text{ }}growth{\text{ }}in{\text{ }}control{\text{ }} - {\text{ }}colony{\text{ }}growth{\text{ }}in{\text{ }}treatment{\text{ }}}}{{colony{\text{ }}growth{\text{ }}in{\text{ }}control}} \times 100$$

The colonies were further observed for following interactional phenomenon given by Rayner [25]:

- Merging of mycelia
- Replacement (growth of one fungus over another)
- Dead lock (in which neither of the fungus was able to grow past another)
- Antagonism (formation of inhibition zones)

### Bulb rot development due to individual, multiple inoculation of pathogens under in vitro conditions and rotting severity

The experiment consisted of five treatments including control. Per treatment eight replications were kept. For individual inoculations, the bulbs were individually inoculated by their respective test pathogens. The bulbs were first surface sterilized by giving a 3-minute dip in 0.1 per cent aqueous mercuric chloride followed by five washings in sterile distilled water. On each bulb injuries were given by removing a tissue plug (5 mm, diameter) with the help of sterilized cork borer and then inoculated with the mycelial disc (5 mm, diameter) of 10 days old test pathogens (*F. solani, P. chrysogenum, B. tulipae* and *A. niger*) [25]. The bulbs kept for control were inoculated with agar plug. The bulbs were incubated at 25 ± 2^o^C for 40 days and were continuously monitored for the following observations.

- Appearance of initial symptoms
- Type of symptoms and lesion size
- Days to partial rot
- Days to final rot
- Rotting severity

For multiple inoculations, the methodology adopted for grouping the bulbs was same as in vitro studies on PDA. The experiment consisted of 12 treatments including control. Per treatment three replications were kept. The pathogens were inoculated on the opposites sides of bulb for dual interaction studies, whereas for triple pathogen interactions, pathogens were inoculated in a triangular fashion, equidistant from each other. In case of interactions involving four pathogens, they were inoculated at four different edges of the bulb. The bulbs were continuously monitored for following observations.

- Type of interaction (Antagonism/ Synergism)
- Days to partial rot
- Days to final rot
- Rotting severity

#### Rotting severity

The rotting severity in case of individual and mixed inoculation of pathogen was calculated by using the following formula:

$$\text{Disease}{\rm{ }}\,\text{severity}{\rm{ }}\left( \% \right){\rm{ }} = \;\;\frac{{\Sigma {\rm{ }}\left( {\text{n} \times \text{v}} \right)}}{{\text{N} \times \text{G}}} \times {\rm{ }}100$$

Where n = number of bulbs affected by disease, v = disease rating (0–3), N = total number of bulbs examined, G = maximum grading.

The rotting severity was determined using modified scale from 0 to 3 described by Chung [26].

## Results

### Isolation and frequency of pathogens associated with bulb rot of Tulip

During the present investigations four pathogens *viz. F. solani, P. chrysogenum, B. tulipae* and *A. niger* were isolated from the diseased bulbs showing symptoms like discoloration which ranged from yellow to brown. Small yellow colour flecks were seen on the outer surface of bulbs which gradually became sunken. The spots on the bulb later coalesced gradually leading to rotting with the emission of a foul odor. Under field conditions the foliage turned purplish in colour and flowers if produced were blasted or deformed (Fig. 1). The isolation results also yielded some other unidentified fungi whose identification couldn’t be ascertained either due to lack of formation of spores or lack of proving pathogenicity tests. *A. niger* was isolated with the highest frequency (40.90%), followed by *F. solani* (30.90%). *B. tulipae* (3.63%) was isolated with the lowest frequency followed by *P*. *chrysogenum* (15.48%) (Table 1).

**Table 1:** Isolation frequency (%) of fungi associated with the bulb rot of tulip during storage conditions

Isolated fungus	Number of Isolates	Isolation Frequency (%)
*Fusarium solani*	34	30.90
*Penicillium chrysogenum*	17	15.48
*Botrytis tulipae*	4	3.63
*Aspergillus niger*	45	40.90
Un identified	10	9.09
Total	110	100

### Pathogenicity tests

**Fig. 2:**
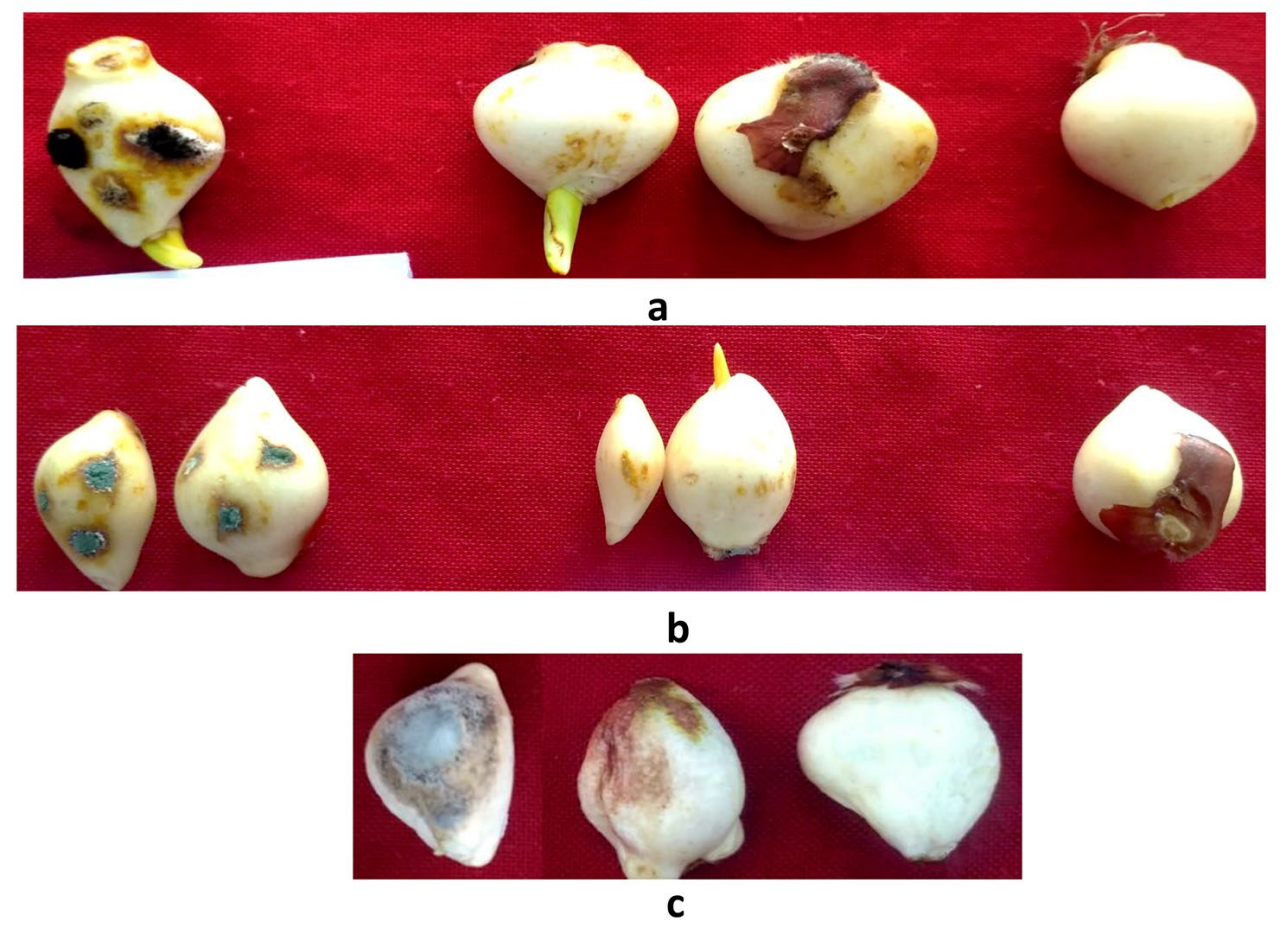
Pathogenicity test of different pathogens associated with the bulb rot of tulip on variety Orange-Emperor using pin-prick method under laboratory conditions. (**a**) Pathogenicity test of *A. niger* The injured (left) and uninjured (middle) were dipped in spore suspensions of *A. niger*. The control (right) was dipped in sterilized water. (**b**) Pathogenicity test of *P. chrysogenum* The injured (left) and uninjured (middle) were dipped in spore suspensions of *P. chrysogenum*. The control (right) was dipped in sterilized water. (**c**) Pathogenicity test of *B tulipae*. The uninjured (left) and injured (middle) were dipped in spore suspensions of *B. tulipae.* The control (right) was dipped in sterilized water

In vitro pathogenicity tests were performed for the frequently isolated fungi viz. *F. solani*, *P. chrysogenum*, *B. tulipae*, and *A. niger* on Orange Emperor variety of tulip following the pin prick method. (Fig. 2). *F. solani* was seen to be least virulent among all the four pathogens with initial symptoms in case of injured ones were produced after 7–8 days and in uninjured ones after 10–11 days [1]. The initial symptoms in case *P. chrysogenum* were seen 7 DAI in injured ones while in case of uninjured were seen 10 DAI. *Botrytis tulipae* was seen to be most virulent among all the bulb rotting pathogens producing symptoms in case of *injured* bulbs 4 DAI while as uninjured showed 6 DAI. Similarly, the plants inoculated with *A. niger* showed symptoms 5 DAI while as the uninjured set showed symptom appearance after 7 days. Nature of symptoms and bulb rot development is elaborated in bulb rot development due to individual inoculation and in Table 2. All the four fungi proved pathogenic to tulip bulbs and completely resembled the original inoculated fungi in their morphological, cultural and pathogenic characteristics, so satisfied Koch’s postulates.

**Table 2:** Bulb rot development under in vitro conditions due to individual inoculation of bulb rotting pathogens

Treatment	Type of symptoms	Days to the appearance of Initial symptom	Lesion size (dia in mm*)	Days to partial rot (range)	Days to final rot (range)	%Rotting Severity*
*Fusarium solani*	Yellow to very light brown coloration around the inoculation site with little mycelial growth visible.	4–5	7	19–20	No complete rotting	42.33
*Penicillium chrysogenum*	Light brown coloration around the inoculation site with slight mycelial growth showing prominent sporulation after 4 days.	3–4	8.5	13–15	No complete rotting	62.33
*Botrytis tulipae*	Dark brown color spots around the inoculation site which coalesced together and made an obavate lesion all aver bulb with raised spots.	2	15	11–12	17	90
*Aspergillus niger*	Yellow to olive green coloration around the inoculation site with white mycelial growth which showed prominent sporulation after 3 days and gave the bulb black sooty appearance	2	10	15–16	20–22	78.33
Control	-	-	-	-	-	7

*Denotes average

### Morphological, cultural and molecular characterization

**Fig. 3:**
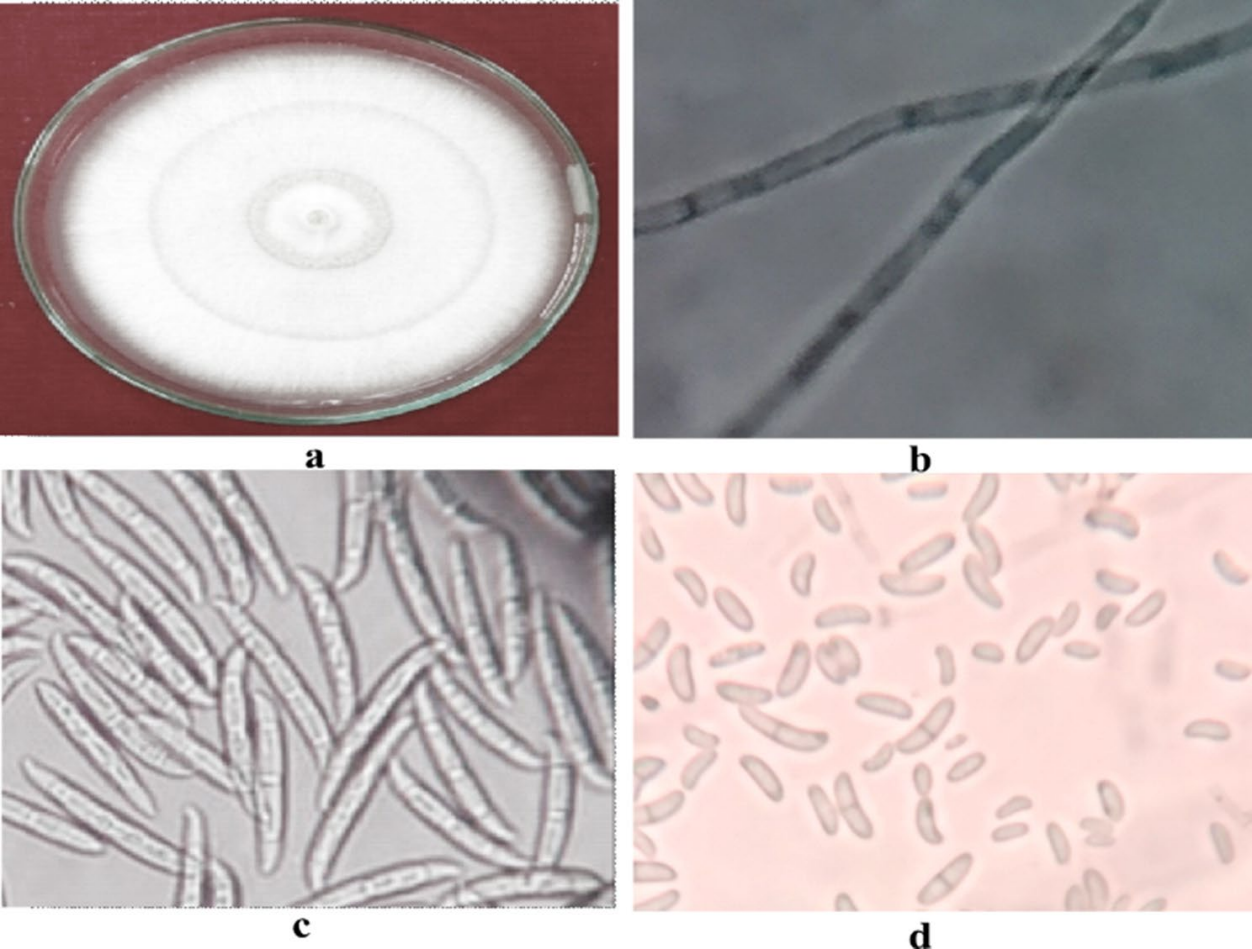
Morpho-cultural characterization of *Fusarium solani* (Mar.) Sacc., associated with bulb rot of tulip (**a**) Colony, (**b**) Septate Mycelium, (**c**) Macroconidia, (**d**) Microconidia

The morpho-cultural as well as molecular identification of the pathogens was performed to authenticate the results. The colony produced by *F. solani* was floccose with a creamish exudation in the rings produced in the culture. Micro conidia were cylindrical to oval frequently septate whereas macro conidia were fusiform (Fig. 3) [1].

**Fig. 4:**
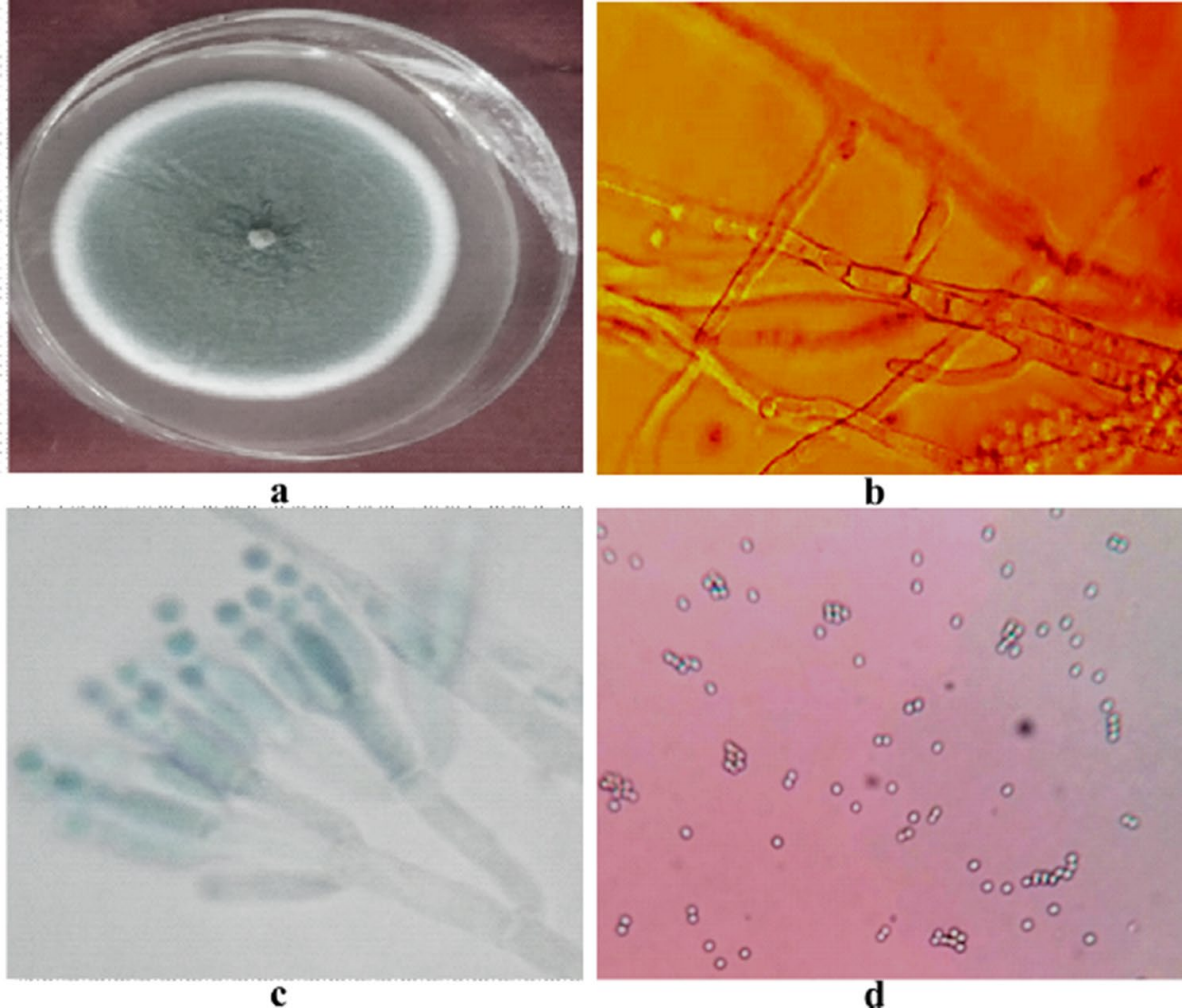
Morpho-cultural characterization of *Penicillium chrysogenum* associated with bulb rot of tulip (**a**) Colony, (**b**) Septate Mycelium, (**c**) Conidiophore (**d**) Conidia

The colonies of *P. chrysogenum* were smooth floccose-fasciculate initially white which later turned to green and velvety with heavy sporulation. The exudation of tiny yellow gel like droplets was seen on the culture plate. Mycelium was smooth and branching, ranging from 3 to 4 μm in width and conidiophores were broom shaped and terverticillate. Conidia were globose-sub globose ranging from 3 to 5 μm (Fig. 4). The pathogen covered the 90 mm dia of petri-plate in 13 days.

**Fig. 5:**
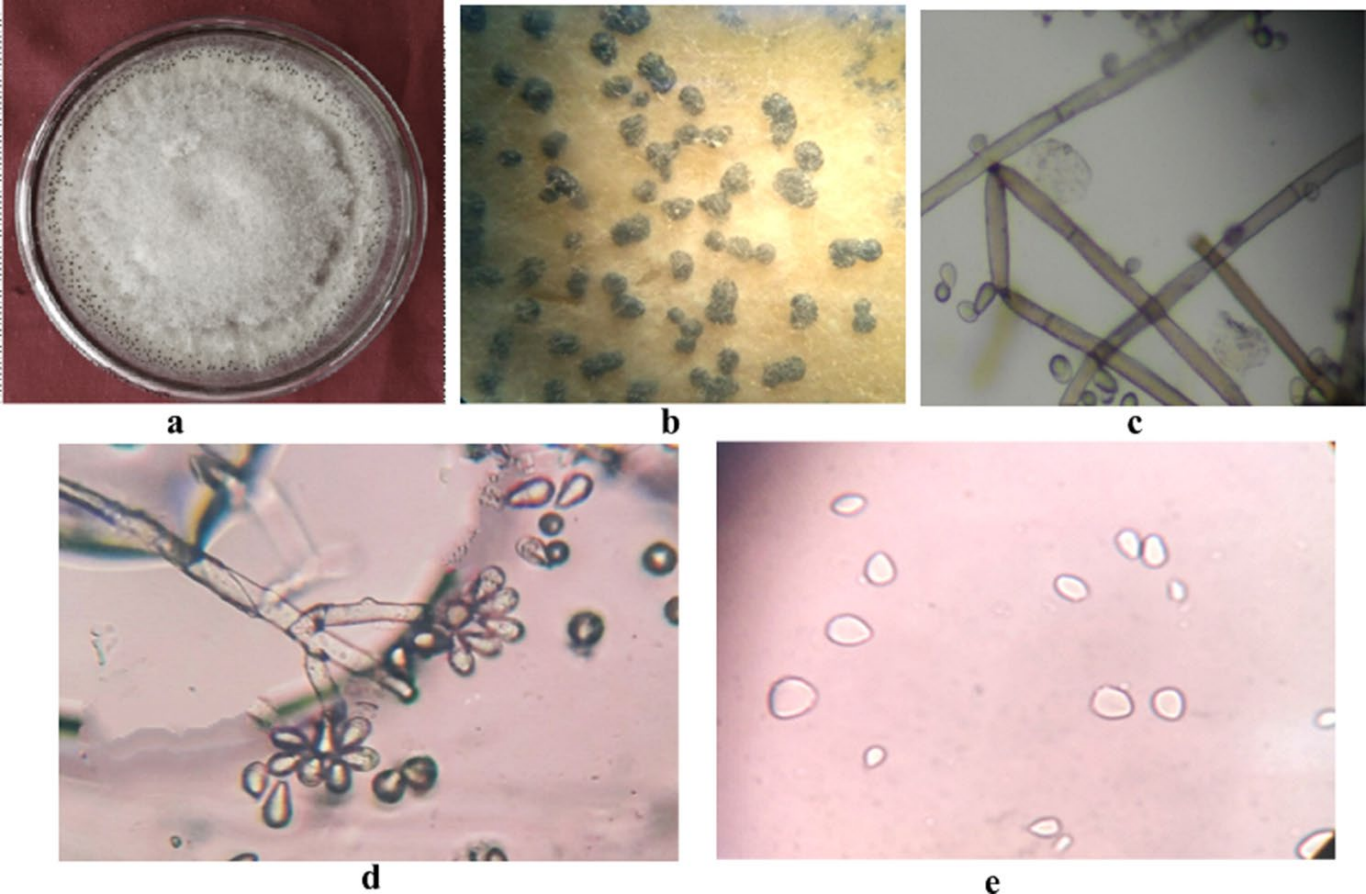
Morpho-cultural characterization of *Botrytis tulipae* (Lib.) Comb. Nov associated with bulb rot of tulip (**a**) Colony, (**b**) Sclerotia, (**c**) Septate Mycelium, (**d**) Conidiophore, (**e**) Conidia

The colony formed by *B. tulipae* on media was fluffy, cotton like greyish in color with aerial tufts of mycelium (Fig. 5). The pathogen covered the entire Petri plate (90 mm dia) in 10 days. The sclerotia produced were shiny, tiny black ranging from 1 to 1.5 mm in size, irregular in outline (loaf shaped) embedded in mycelia, formed in concentric rings in the plate. Mycelium was hyaline to brownish, septate, branched measuring 7.50–15.0 μm in width. The conidiophores were branched, septate, hyaline to brownish in color resembling a tree or grape like structure which measured 27.5–35.0 × 14.5-17.0 μm. The conidia were single celled, obovate, hyaline to brownish in colour, ranging from 6.8 - 12.0 × 5.0 -8.5 μm born terminally in clusters on conidiophores.

**Fig. 6:**
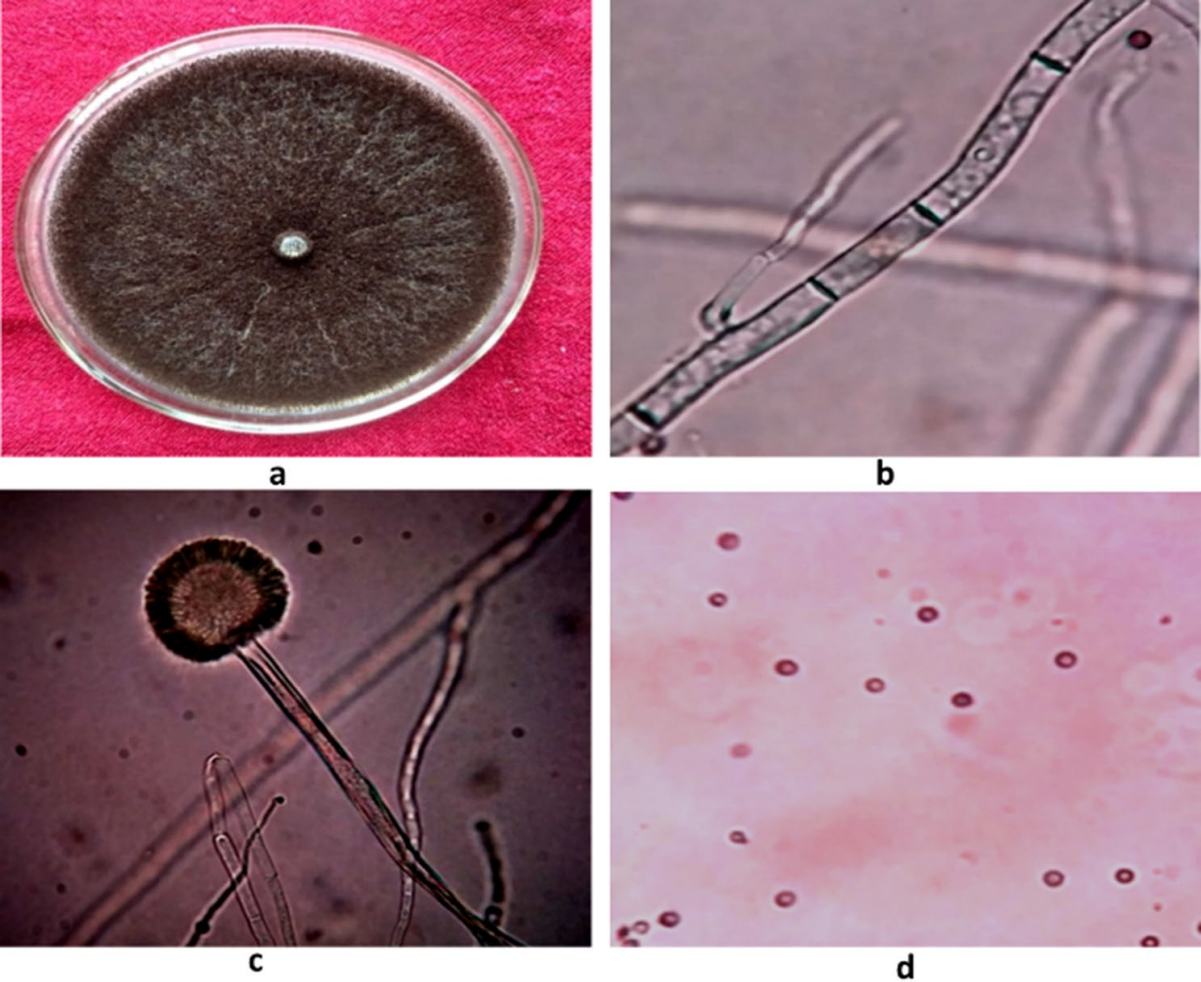
Morpho-cultural characterization of *Aspergillus niger* Gams associated with bulb rot of tulip (**a**) Colony, (**b**) Septate Mycelium, (**c**) Conidiophore, (**d**) Conidia

In case of *A. niger* the colony formed on media was granular, fast growing with light yellow periphery which later turned dark black, with profuse growth and heavy sporulation. The pathogen covered 90 mm of Petri plate in 5–6 days (Fig. 6). Mycelium was septate, branched, brownish-black in colour which measured 2.0 -3.4 μm in size. Conidiophores were radiate, bi-seriate, brownish-black in colour which measured 3.4-6.0 μm in width and bore conidia which were globose-subglobose, single celled, brownish- black in colour measuring 4.0–5.0 μm.

### Molecular Characterization and phylogeny

The genomic DNA was extracted from fungal cultures and loaded on 0.7% agarose gel that was run for 30 min. The DNA extracted from different pathogens resulted in single high molecular weight intact bands, indicating a good quality DNA. PCR amplification under standard conditions was carried out, and the amplified products were run on 1% agarose gel. PCR showed the amplified product size in the range of ∼500–550 bp for ITS1 and ITS4 region for different pathogens and PCR product of *β-tubulin* resulted in amplicon size of ∼550 bp (Table 3). After sequencing the PCR product and analyzing the results with BLASTn, all sequences of the respective pathogens showed 98-100% sequence homology with GenBank sequences. All the pathogens were subsequently identified on the basis of molecular identification and the sequences identified from Nucleotide Sequencing and bioinformatic analysis were successfully published in GenBank (www.ncbi.nlm.in). This is the first report of *A. niger* and *P. chrysogenum* associated with the bulb rot of tulip in India. The accession numbers of the identified pathogens are given in Table 3.

**Table 3:** Pathogens identified through DNA sequencing

S. No	Gene	Pathogens	Amplicon size	GenBANK Accession No’s
1	ITS	*Penicillium chrysogenum*	550	OP393913
2	ITS	*Botrytis tulipae*	500	MN633367
3	ITS	*Aspergillus niger*	550	MN633366
4	*β-tubilin*	*Penicillium chrysogenum*	550	OP410742
5	*β–tubulin*	*Aspergillus niger*	550	OP410743

**Fig. 7:**
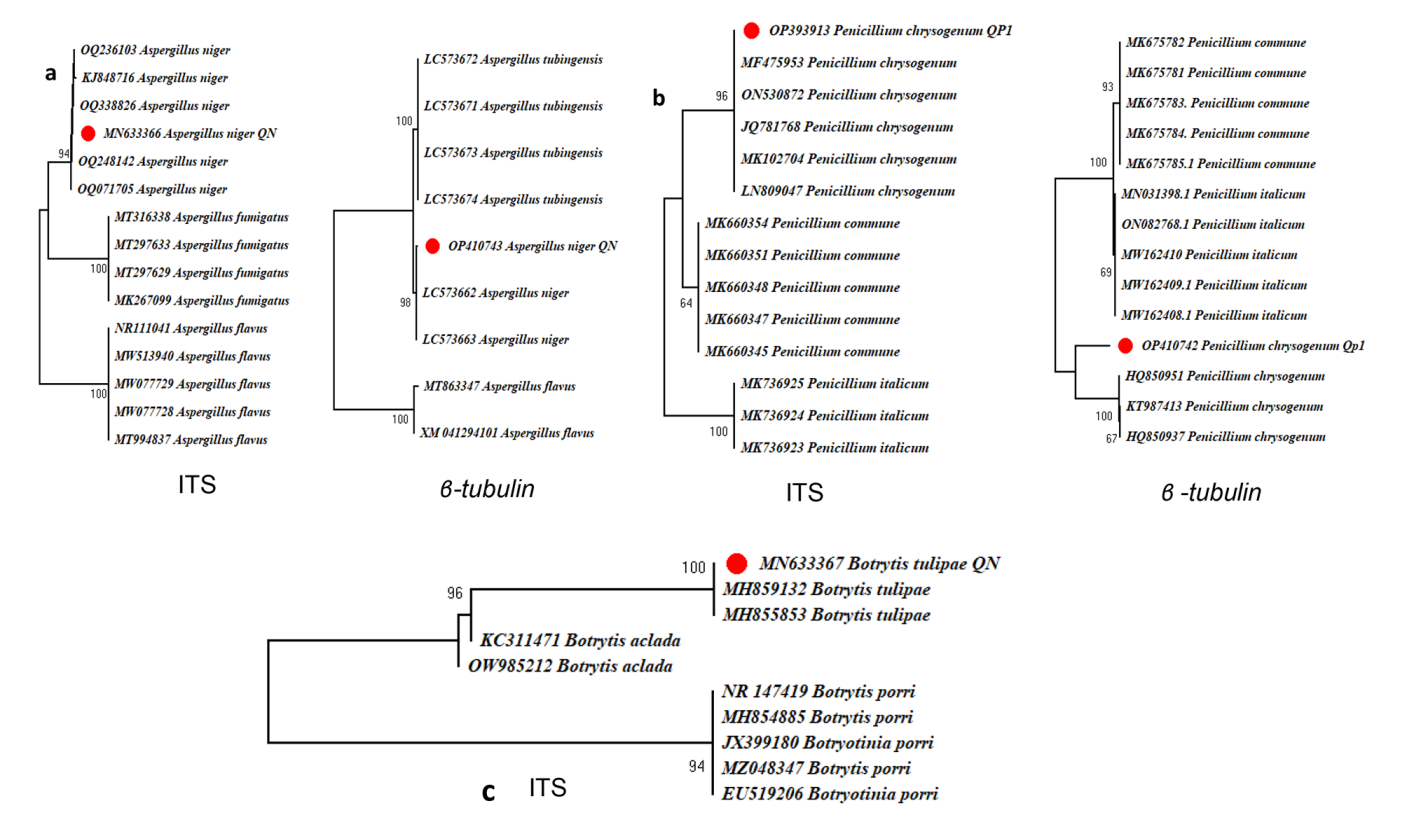
(**a**) Phylogenetic analysis of *A. niger* by neighbour joining method based on ITS sequences and *β-tubulin* sequences. The position of original isolate is depicted in red (**b**), Phylogenetic analysis of *P. chrysogenum* by neighbour joining method based on ITS sequences and *β-tubulin* sequences. The position of original isolate is depicted in red (**c**), Phyllogenetic analysis of *B. tulipae* by neighbour joining method based on ITS sequences. The position of original isolate is depicted in red

An optimal dendrogram was constructed using MEGA-X software V-10.0, and different taxa were clustered together in a bootstrap test 1000 replicates using sequences of ITS region, and *β-tubulin* genes that were compared with their respective hits retrieved from NCBI database and compared with the already available sequences. Phylogenetic analysis revealed that our isolates collected from Kashmir region clustered along with other submitted *Penicillium, Botrytis* and *Aspergillus* isolates from GenBank. (Fig. 7). Our isolate of *P. chrysogenum* showed sequence homology with the isolates from china and Egypt. *A. niger* clustered with the isolates of Egypt and Japan. Similarly *B tulipae* showed similarity with the isolates of Netherlands.

### In vitro interaction of pathogens associated with the bulb rot of tulip on potato dextrose agar medium

**Fig. 8:**
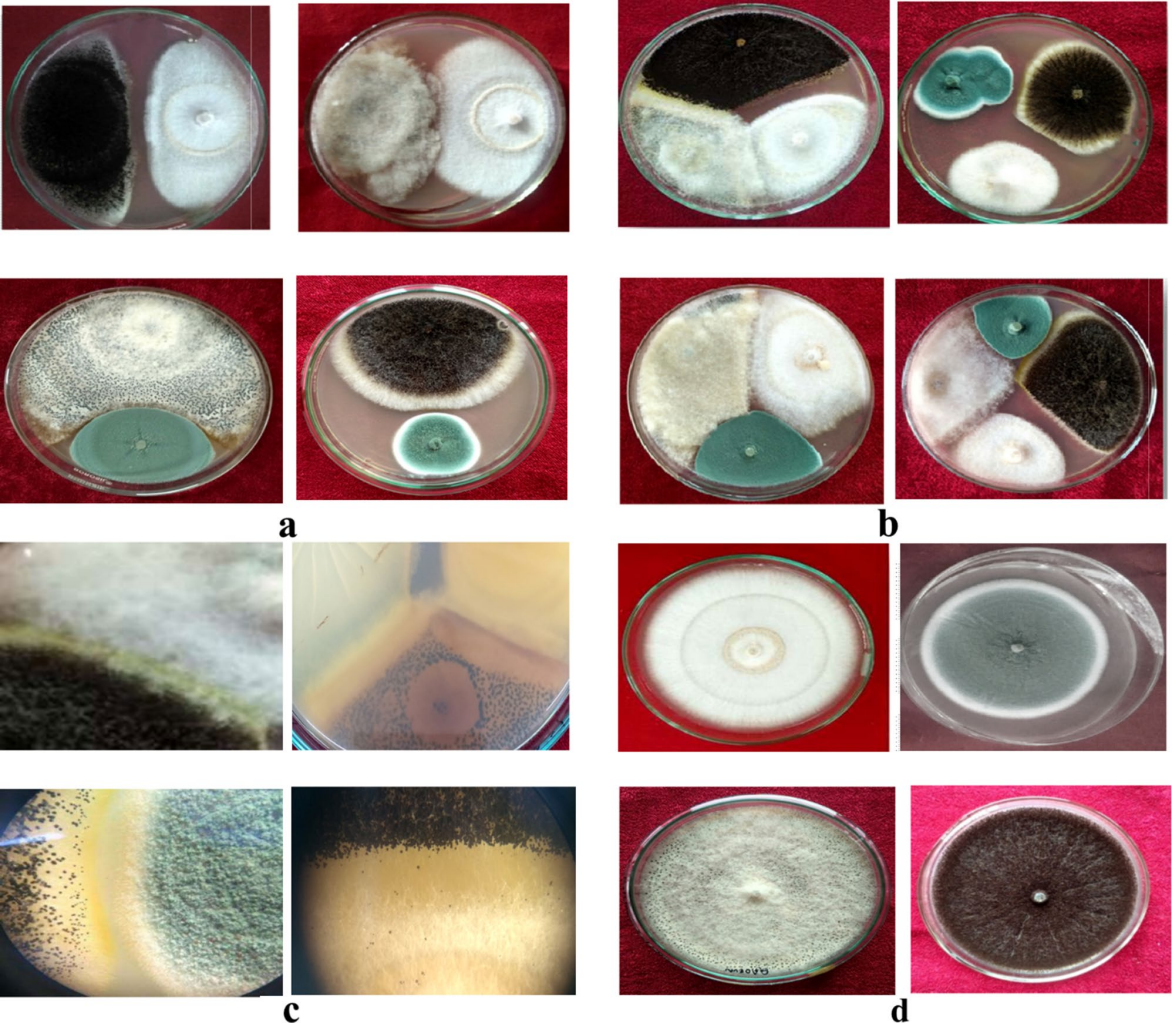
Pathogen-Pathogen interaction of different pathogens associated with bulb rot of tulip on PDA. (**a**) Pathogens combined under dual culture opposite to one another, (**b**) Pathogens combined in three and four combinations, (**c**) Nature of different interactions like dead lock with the formation of yellow colored zones between *A. niger* and *B. tulipae*, formation of profused sclerotial bodies by *B. tulipae* when co-cultured with other pathogens, antibiosis between *A. niger* and *P. chrysogenum* (**d**) Single culture of *F. solani*, *P*. *chrysgenum*, *B. tulipae* and *A. niger* which served as control

Perusal of the data presented in Table 4 indicated that in case of dual culture of *F. solani* with *P. chrysogenum* or *A. niger*, an inhibition zone of 5.00 and 8.50 mm (Fig. 8), respectively was formed at the zone of interaction indicating antibiosis. However, there was merging of mycelium between *F. solani* and *B. tulipae* and no inhibition zone was formed. In triple interaction studies, the highest mycelial inhibition of 71.22 per cent was recorded when *F. solani* was grown along with *A. niger* and *B. tulipae* followed by 63.33 per cent in case of *P. chrysogenum* and *A. niger* and least (62.31%) when *F. solani* was grown in combination with *P. chrysogenum* and *B. tulipae*. The average per cent mycelial inhibition of *F. solani* was 73.33 per cent when all bulb rotting pathogens were inoculated simultaneously. The mycelium of *F. solani* merged with the mycelium of *B. tulipae* but showed antibiosis with *A. niger* with an inhibition zone of 11.50 mm (Table 4).

**Table 4:** Pathogen-Pathogen interaction of different pathogens associated with bulb rot of tulip on PDA.

Treatment	Type of interaction	Average Radial growth covered (mm)	Percentage mycelial inhibtion*	Inhibition zone* (mm)
*Fusarium solani vs. Penicillium chrysogenum*	Antibiosis	46.00	48.88	5.00
*Fusarium solani* vs. *Botrytis tulipae*	Merging	43.00	52.22	0.00
*Fusarium solani vs. Aspergillus niger*	Antibiosis	41.00	54.11	8.50
*Penicillium chrysogenum vs. Botrytis tulipae*	Deadlock	35.20	61.11	0.00
*Penicillium chrysogenum vs. Aspergillus niger*	Antibiosis	30.00	66.66	5.40
*Botrytis tulipae vs. Aspergillus niger*	Deadlock	38.00	57.49	0.00
*Fusarium solani vs. Penicillium chrysogenum vs. Botrytis tulipae*	Deadlock^FP^, Merging^FB^	33.5	62.77	0.00
*Fusarium solani vs. Penicillium chrysogenum vs. Aspergillus niger*	Antibiosis^FP^ Antibiosis^FA^	33.00	63.33	5.00^FP^ 11.50^FA^
*Fusarium solani vs. Aspergillus niger vs. Botrytis tulipae*	Antibiosis^FA^, Merging^FB^	25.00	71.22	11.50^FA^
*Botrytis tulipae vs. Penicillium chrysogenum vs. Aspergillus niger*	Deadlock^BP^, Deadlock^BA^,	44.50	50.55	0.00
*Fusarium solani vs. Penicillium chrysogenum vs. Botrytis tulipae vs. Aspergillus niger*	,Merging^FB^, Antibiosis^FA^	24.00	73.33	11.50^FA^
Control				

F, P, B and A denote *Fusarium solani, Penicillium chrysogenum, Botrytis tulipae* and *Aspergillus niger*, respectively

*Denotes average

The data presented in Table 4 revealed that the interaction of *P. chrysogenum* with the other bulb rotting pathogens significantly inhibited the mycelial growth of *P. chrysogenum*. When *P. chrysogenum* was paired with *A. niger* there was maximum inhibition of mycelial growth (66.66%) followed by *B. tulipae* (61.11%). The least mycelial inhibition was observed when *F. solani* was paired with *P. chrysogenum* in dual culture. An analysis of the interactional phenomenon revealed that antibiosis existed between *P. chrysogenum* and *F. solani*, *P. chrysogenum* and *A. niger* while deadlock was observed between *P. chrysogenum* and *B. tulipae* (Fig. 8).

Deadlock with the formation of yellow color contact zone was seen when *B. tulipae* was paired with *A. niger* whereas only deadlock was seen when *B. tulipae* was paired with *P. chrysogenum*. Antibiosis was seen when *A. niger* was paired with *F. solani* and *P. chrysogenum* with an average inhibition zone of 8.50 mm and 5.40, mm respectively.

### Bulb rot development due to individual, multiple inoculation of pathogens under in vitro conditions and rotting severity

**Fig. 9:**
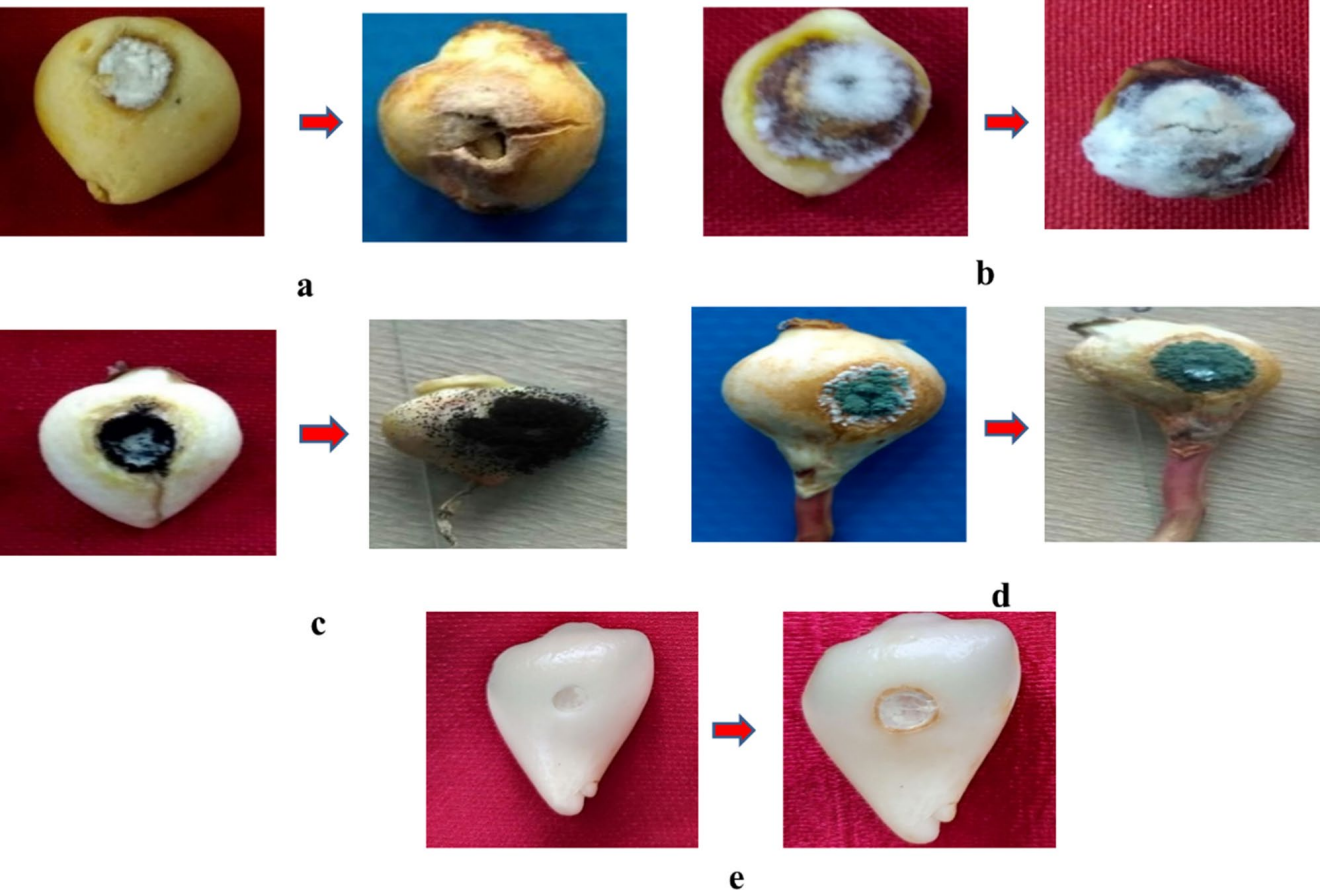
Bulb rot development under in-vitro conditions due to individual inoculation of bulb rotting pathogens using cork borer method. The control was inoculated with clean agar plug. *B. tulipae* was the most virulent and *F. solani* the least virulent. (**a**) Symptom appearance (left) and Bulb rot development (right) due to inoculation of *F. solani*, (**b**) Symptom appearance (left) and Bulb rot development (right) due to inoculation of *B. tulipae*, (**c**) Symptom appearance (left) and Bulb rot development (right) due to inoculation of *A. niger*, (**d**) Symptom appearance (left) and Bulb rot development (right) due to inoculation of *P. chrysogenum*, (**e**) Control: At the time of initial inoculation (left), final (Right).

**Fig. 10:**
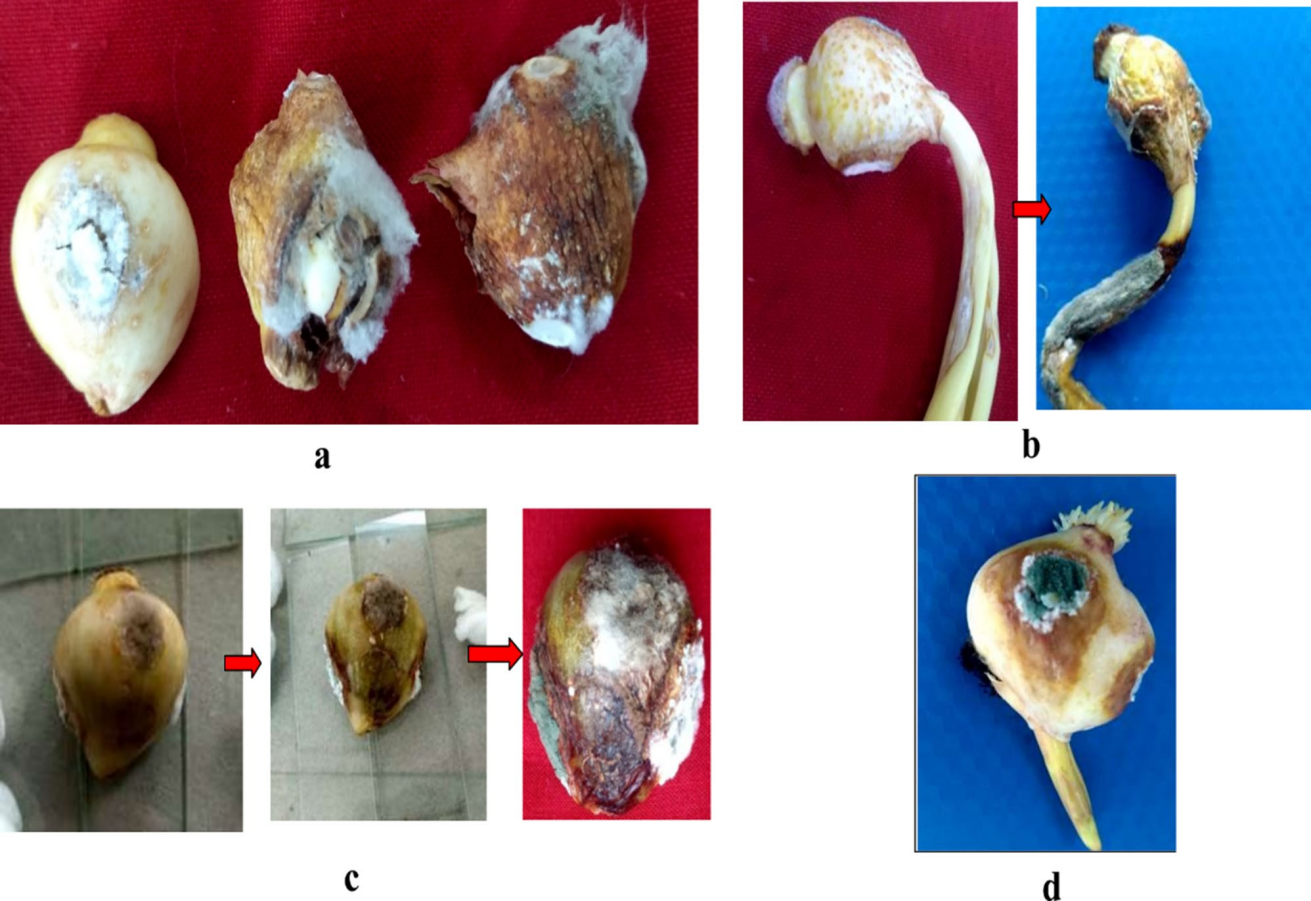
Bulb rot development under in-vitro conditions due to co-inoculation of multiple pathogens using cork borer. (**a**) Bulb rot development due to inoculation of *F. solani* (left), *B. tulipae* alone (middle), representing more rotting severity, *F. solani* and *B. tulipae* in combination (right) representing partial rot (**b**) Bulb rot development due to co- inoculation of *F. solani* and *P. chrysogenum*: initial rot (left) partial rot (right) within 18 days, (**c**) Bulb rot development due to co-inoculation of *F. solani*, *P. chrysogenum* and *B. tulipae*: Initial rot (left), partial rot within 24 days (middle), final rot (left) representing complete rot within 35 days, (**d**) Bulb rot development due to co-inoculation of *F. solani*, *P. chrysogenum* and *A. niger* representing partial rot

The results are presented in Tables 2 and 5; Figs. 9 and 10. When pathogens were individually inoculated with different bulb rotting pathogens the initial symptoms were first recorded in case of *B. tulipae* and *A. niger* (2 days) followed by *P. chrysogenum* (3–4 days) and *F. solani* (4–5 days).

**Table 5:** Bulb rot development under in vitro conditions due to interaction of multiple bulb rotting pathogens

Interactions	Type of interactional phenomenon	Days to partial rot	Days to complete rot	%Rotting Severity*
*Botrytis tulipae* vs. *Penicillium chrysogenum*	Synergism	10	14	83.66
*Botrytis tulipae* vs. *Aspergillus niger*	Synergism	11	17	77.00
*Aspergillus niger* vs. *Penicillium chrysogenum*	Synergism	13	19	73.66
*Fusarium solani* vs. *Penicillium chrysogenum*	Antagonism	18	No complete rotting	62.33
*Fusarium solani* vs. *Aspergillus niger*	Antagonism	20	No complete rotting	55.00
*Fusarium solani* vs. *Botrytis tulipae*	Antagonism	20	No complete rotting	42.33
*Botrytis tulipae* vs. *Aspergillus niger* vs. *Fusarium solani*	Antagonism	30	40	76.00
*Botrytis tulipae* vs. *Penicillium chrysogenum* vs. *Aspergillus niger*	Antagonism	12	No complete rotting	60.00
*Fusariumsolani* vs. *Botrytis tuliapae* vs. *Penicillium chrysogenum*	Antagonism	24	35	85.66
*Aspergillus niger* vs. *Penicillim chrysogenum* vs. *Fusarium solani*	Antagonism	17	No complete rotting	63.66
*Fusarium solani* vs. *Penicillium chrysogenum* vs. *Botrytis tulipae* vs. *Aspergillus niger*	Antagonism	12	No complete rotting	68.00
Control	-	-	-	7

*Denotes average

The average lesion size recorded was maximum in *B. tulipae* (15 mm) followed by *A. niger* (10 mm) followed by *P. chrysogenum* (8.5 mm) and the least was seen in *F. solani* (7 mm). *B tulipae* was able to cause complete rot of tulip bulb in 17 days followed by *A. niger* (20–22) days where as *P. chrysogenum* and *F. solani* did not cause the complete rot f tulip bulb.

The type of symptoms varied when tulip bulbs were inoculated with different bulb rotting pathogens. When tulip bulbs were inoculated with *F. solani* there was yellow to very light coloration around the inoculation site with little dull whitish mycelial growth visible after six days of inoculation whereas when bulbs were inoculated with *P. chrysogenum* there was light to brown colouration around the inoculation site with slight mycelial growth which showed sporulation after four days. *B. tulipae* showed the most aggressive symptoms as compared to all the four rotting pathogens. The symptoms produced were dark brown with raised spots which made an obovate lesion around the inoculation site and later caused the complete decay of bulb. In case of *A. niger* there was yellow to olive green coloration around the inoculation site with white mycelial growth which showed prominent sporulation after three days and gave the bulb black sooty appearance.

In case of multiple inoculation of pathogens, there was synergistic interaction when *B. tulipae* was paired with *P. chrysogenum* which took ten days to partial rot and fourteen days to complete rot (Table 5). Similarly, synergism also existed when *B. tulipae* was co inoculated with *A. niger* which took 11 days to initial rot and 17 days to completely rot the bulb and when *A. niger* was paired with *P. chrysogenum* on tulip bulb the bulb was partially rotted in 13 days and completely rotted in 19 days. All other combinations of bulb rot pathogens showed potential degree of antagonism and increased the number of days for partial as well as complete rot. The bulbs showed complete rotting when *F. solani* was combined with *B. tulipae* and *P. chrysogenum* and rotted the bulb in 35 days where as when *B. tulipae* was combined with *A. niger* and *F. solani* it took 30 days to partial rot and 40 days to complete rot. No complete rot was observed in the rest of other combinations (Table 5).

#### Rotting severity

The severity of rotting caused by the inoculation of individual and multiple inoculation of pathogens is shown in Figs. 9 and 10; Tables 2 and 5. In case of individual inoculations, the average rotting severity was maximum when tulip bulbs were inoculated with *B. tulipae* (90%) followed by *A. niger* (78.33%) and *P. chrysogenum* (62.33%). However *F. solani* (42.33%) caused the least rotting severity compared to other pathogens (Table 2).

In case of multiple inoculations, the data presented in Table 5 revealed that the maximum rotting severity was seen when *F. solani* was paired with *B. tulipae* and *P. chrysogenum* (85.66%) followed by *B. tulipae vs. P. chrysogenum* (83.66%) and *B. tulipae vs. A. niger* (77%). Co-inoculations of *B. tulipae vs. A. niger vs. F. solani* resulted in 76% rotting severity, whereas, the least rotting severity of 42.33% was oserved in co-inoculations with *F. solani vs. B. tulipae* followed by 55% rotting severity caused by co-inoculating *F. solani and A. niger.*

## Discussion

Tulip, one of the most important floricultural crops under the temperate conditions of Kashmir valley, occupies a prominent status in Kashmir in terms of tourism and in turn economy of our state. It is affected by various diseases in field as well as in storage. However, bulb rot directly limits its profitable cultivation. Bulb rot is a serious problem in various bulbous crops like Tulip, Lilium, Onion, garlic etc. A large collection of pathogens belonging to different genera are responsible to cause rot in them among which the notable genera are *Fusarium, Penicillium* and *Aspergillus.* In our study *A. niger* was isolated at higher frequency than other bulb rot pathogens. This can be attributed to the fact that it is highly sporulating pathogen and is most predominant one during storage conditions, responsible for causing rots of a number of crops [27, 28, 29].

The morpho-cultural as well as molecular identification of the pathogens was performed to authenticate the results. The morphological characteristics of *A. niger*, *P. chrysogenum* and *B. tulipae* were confirmed with the standard manuals and studies performed earlier on the pathogens [30–32]. In case of *A. niger* and *P. chrysogenum* in addition to ITS region we sequenced the additional βtubulin gene to verify the identification as explained by Glass and Donaldson in 1994 for the identification of filamentous ascomycetes. The morpho-cultural description of *B. tulipae* is beautifully decribed by Hopkins in 1921 [33] and our results were in clear resemblance with him. Also the first report of this pathogen from India is from the Kashmir region which supports our findings [34]. Additionally, molecular identification using ITS region was done to authenticate the results which further clarified the identification processes. These results are in agreement with a study conducted by Erper while working on *B. tulipa* [31].

Since more than one causal pathogen was isolated from the diseased bulbs, it was desirable to study the interactional phenomenon between the isolated fungi which could finally help in designing the effective disease management strategies. Pasteur is credited with making the first observations about microbial communities as disease-causing agents in the 1800s. He noted that a disease might result from the cooperative or competitive interactions of many microorganisms [35]. To study the interactional phenomenon between the pathogens, the pathogens were first inoculated on artificial media PDA. Similar procedure was adopted by Rasiukevičiūtė in 2017 for studying the mycelial compatibility of *Botrytis cineria* isolates originated from apple and strawberry in Lithuania [36]. Various types of interactional phenomenon like antibiosis, deadlock, merging of mycelia was seen between the associated bulb rotting pathogens. These kinds of interactions between the pathogens suggest a potential degree of antagonism between the pathogens. In addition to these interactions production of seclerotial bodies in only five-day old culture of *B. tulipae* clearly indicates the stress state of *B*. *tulipae* as a result of antagonistic effect of other bulb rotting fungi. Antibiosis between *F. solani* and *A. niger*, *F. solani* and *P. chrysogenum*, merging of mycelia between *F. solani* and *B tulipae*, these profound antagonistic interactions observed on Petri plates can well be co-related to antagonistic nature of interaction showed by the pathogen on their natural host. Since this is the first interactional study carried out on the bulb rot of tulip and with the experimental methodology followed to perform the present study it was not possible to determine what chemicals or metabolites are secreted by these pathogens responsible for showing antibiosis. Although evidence does occur for metabolites and toxins secreted by different species of *Fusarium*, *Aspergillus* and *Penicillium* that are antagonistic to sensitive pathogens within the host and may lead to these possible interactions between the pathogens [37, 38]. Furthermore, it is pertinent to mention here that the role of *P. chrysogenum* as producer of the beta-lactam antibiotic penicillin which might be the reason of antagonism shown by the pathogen in the vicinity of other pathogenic fungi [39]. In the same way the cocktail of secondary metabolites produced by *A. niger* might be the reason for the possible interactions as witnessed by Chatterjee [40] while working on the interaction studies of *Aspergillus niger, Fusarium verticillioides* and *Clonostachys rosea* in a mixed culture. Further studies need to be done on this topic to get the deeper knowledge of this interface and verify the results.

Among the four pathogens *B. tulipae* was reckoned to be the most virulent one which was clearly shown by the average lesion size of 15 mm, appearance of early symptoms (2 days), and its tendency to cause complete rotting when inoculated individually on tulip bulbs. In addition, *B. tulipae* was able to cause the maximum rotting severity followed by *A. niger*. However, the results varied when same pathogens were inoculated on tulip bulbs in different combinations. Out of eleven combinations, eight interactions showed antagonism and three interactions viz. *B. tulipae* vs. *P. chrysogenum*, *B. tulipae* vs. *A. niger*, *A. niger* vs. *P. chrysogenum* showed synergism. Of the combinations which showed antagonism, only two viz. *B. tulipae* vs. *A. niger* vs. *F. solani* and *F. solani* vs. *B. tulipae* vs. *P. chrysogenum* caused complete rotting of bulb although the time required was considerably more than the individual inoculations. The other combinations in which mixed infection was involved caused partial rotting of the bulb. These results are in confirmation with various researches done on other crops like Santamaria [41] used the same inoculation technique on red and jack pine seedlings and observed the reduction of symptom severity caused by *Diplodia pinea* in presence of *Diplodia scrobiculata* in pine seedlings. *Fusarium oxysporum* (Fo47) and *Fusarium oxysporum* (Fol8) in tomato showed less disease severity in mixed infection compared to when inoculated individually [42]. Although there have been various reports of antagonistic and synergistic pathogen- pathogen interaction in plant pathology, still the mechanisms underlying these interactions are mostly unknown. In contrast to the single pathogen disease system in which the interactions involve only one host and one pathogen, which decides the level of damage the plant experiences, the “host–multiple pathogen” infection in addition to the “host–pathogen” interaction involves “pathogen–pathogen” interaction, as well as the corresponding host response to this warfare which ultimately decides the fate of the disease [16]. Depending on the genes involved and how they interact when several pathogens attack the same host, the overall severity of the disease may increase or decrease [43, 44]. In the course of our study we draw the speculation that both the synergism and antagonism existed between the pathogens although antagonism served as the dominating interaction (Table 5). We observed cooperation when *B. tulpae* was paired with *P. chrysogenum* which was able to cause the complete rotting in 14 days compared to the most virulent bulb rotting pathogen *B. tulipae* causing rot in 17 days suggesting that in addition to competition, pathogens can also form relationships and cooperate with each other that are crucial for pathogenesis [45]. Cooperation can enhance pathogen persistence by supporting greater reproduction and multiplication rates and there by exaggerating the damage caused by the pathogens. The same synergistic relationship took a different shift when co-infected with the third pathogen *A. niger* and was not able to cause the complete rotting indicating the change in the disease pattern after 12 days of partial rotting (Table 5). Key factors for these shifts are not well studied; although certain changes can be induced by multiple pathogen signals that alter host defence responses and thus alter the virulence pattern [46].

## Conclusion

Kashmir valley occupies a monopolistic position in the tulip production all over India and is one of the important crops in terms of economy of the state. Bulb rot is posing a serious threat to its production and is one of the reasons for the country to import the tulip bulbs from Netherlands. This is the first study carried out on multiple pathogens associated with bulb rot of tulip. We reported *A. niger and P. chrysogenum* as two new pathogens to be associated with the disease. An overall shift in the virulence pattern was observed in the multiple infection disease system revealing the importance of pathogen– pathogen interactions in the field of plant pathology. We conclude that pathogen– pathogen interactions is the key player in shaping the overall picture of the rotting severity. We presented the surface picture of this art work, however the biochemical and molecular studies can add a dimension to the work and further clarify this three-way dialogue.

## Data Availability

The data presented in this study are available on request to the corresponding author. The data are not publicly available due to privacy.
